# Inhibitors of STAT3, β‐catenin, and IGF‐1R sensitize mouse PIK3CA‐mutant breast cancer to PI3K inhibitors

**DOI:** 10.1002/1878-0261.12053

**Published:** 2017-04-06

**Authors:** Vanessa F. Merino, Soonweng Cho, Xiaohui Liang, Sunju Park, Kideok Jin, Qian Chen, Duojia Pan, Cynthia A. Zahnow, Alan R. Rein, Saraswati Sukumar

**Affiliations:** ^1^ Department of Oncology Johns Hopkins University School of Medicine Baltimore MD USA; ^2^ Department of Molecular Biology and Genetics Johns Hopkins University School of Medicine Baltimore MD USA; ^3^ HIV Dynamics and Replication Program National Cancer Institute Frederick MD USA

**Keywords:** breast, IGF, mutation, PIK3CA, STAT3

## Abstract

Although mutations in the phosphoinositide 3‐kinase catalytic subunit (PIK3CA) are common in breast cancer, PI3K inhibitors alone have shown modest efficacy. We sought to identify additional pathways altered in PIK3CA‐mutant tumors that might be targeted in combination with PI3K inhibitors. We generated two transgenic mouse models expressing the human PIK3CA‐H1047R‐ and the ‐E545K hotspot‐mutant genes in the mammary gland and evaluated their effects on development and tumor formation. Molecular analysis identified pathways altered in these mutant tumors, which were also targeted in multiple cell lines derived from the PIK3CA tumors. Finally, public databases were analyzed to determine whether novel pathways identified in the mouse tumors were altered in human tumors harboring mutant PIK3CA. Mutant mice showed increased branching and delayed involution of the mammary gland compared to parental FVB/N mice. Mammary tumors arose in 30% of the MMTV‐PIK3CA‐H1047R and in 13% of ‐E545K mice. Compared to MMTV‐Her‐2 transgenic mouse mammary tumors, H1047R tumors showed increased upregulation of Wnt/β‐catenin/Axin2, hepatocyte growth factor (Hgf)/Stat3, insulin‐like growth factor 2 (Igf‐2), and Igf‐1R pathways. Inhibitors of STAT3, β‐catenin, and IGF‐1R sensitized H1047R‐derived mouse tumor cells and PIK3CA‐H1047R overexpressing human HS578T breast cancer cells to the cytotoxic effects of PI3K inhibitors. Analysis of The Cancer Genome Atlas database showed that, unlike primary PIK3CA‐wild‐type and HER‐2^+^ breast carcinomas, PIK3CA‐mutant tumors display increased expression of AXIN2, HGF, STAT3, IGF‐1, and IGF‐2 mRNA and activation of AKT, IGF1‐MTOR, and WNT canonical signaling pathways. Drugs targeting additional pathways that are altered in PIK3CA‐mutant tumors may improve treatment regimens using PI3K inhibitors alone.

**Abbreviations**
muPIK3CA: mutant PIK3CA
PI3Ki: PI3K inhibitor
PIK3CA: phosphoinositide 3‐kinase catalytic subunit

## 1 Introduction

The phosphatidylinositol 3‐kinase (PI3K) pathway is a key regulator of growth, survival, and metabolism in both normal and malignant cells (Zhao and Vogt, 2008). Over 70% of breast cancers show activation of the PI3K pathway through mechanisms such as HER2 amplification, deletion of the tumor suppressor PTEN, or oncogenic mutations in PIK3CA (Zhao and Vogt, 2008). The PIK3CA gene encodes the catalytic subunit p110α, and its amplification and/or mutation is associated with several types of solid tumors (Bachman *et al*., 2004; Kadota *et al*., 2009; Samuels *et al*., 2004; Wu *et al*., 2005). Activating somatic mutations in PIK3CA are present in ~ 30% of human breast cancers of all stages (Bachman *et al*., 2004; Barbareschi *et al*., 2007; Miller, 2012; Saal *et al*., 2005). In 47% of these cases, mutations occur in the kinase domain, most frequently the H1047R substitution in exon 20. In 33% of these cases, mutations occur in the helical domain; here, the most frequent ones are the E545K and E542K substitutions in exon 9 (Bachman *et al*., 2004; Bader *et al*., 2005). PIK3CA mutations cause a gain of enzymatic function, stimulate AKT signaling, induce growth factor, and anchorage independent growth in culture (Bader *et al*., 2005; Ikenoue *et al*., 2005; Isakoff *et al*., 2005). Expression of H1047R‐mutant PIK3CA in the mammary gland induces tumors in transgenic mice (Adams *et al*., 2011; Meyer *et al*., 2011; Tikoo *et al*., 2012; Yuan *et al*., 2013). Therefore, inhibition of PI3K represents a potentially attractive strategy for the treatment of breast cancer. In fact, a number of PI3K inhibitors (PI3Ki) have entered clinical trials (Zardavas *et al*., 2014).

Laboratory studies and early clinical trials indicate that several of the PI3Ki demonstrate preferential inhibition of tumors with PIK3CA mutations (Mayer *et al*., 2017; O'Brien *et al*., 2010). However, while long‐term stabilization and partial tumor responses are observed in PIK3CA‐positive breast cancers treated with PI3Ki (NCT01219699) (Brana and Siu, 2012), the majority of mutant cancers do not experience substantial regression (Bendell *et al*., 2012). Strategies to overcome both *de novo* and adaptive resistance to PI3Ki have employed combination therapy. Combined inhibition of PI3K and mammalian target of rapamycin complex (mTORC) is associated with stable disease in phase 1 clinical trials (Markman *et al*., 2012; Papadopoulos *et al*., 2014). In addition, clinical trials using a combination of PI3Ki with fulvestrant, IGF‐1R antibody (AMG479), or lapatinib have been initiated (Zardavas *et al*., 2014).

This study sought to characterize the molecular mechanisms of action of mutant p110α in tumors derived from genetically engineered mouse models to further define targets for treating PIK3CA‐driven tumors and their metastatic progression, with the goal of developing mechanism‐based combination therapies.

## 2 Materials and methods

### 2.1 Cell lines and reagents

The HS578T cell line was obtained from the American Type Culture Collection (Manassas, VA, USA). Low passages of the cell line were used to avoid loss of fidelity to the original cells. Stable cell lines were derived from PIK3CA‐H1047R mouse mammary tumors. Cell lines derived from recurrent H1047R mouse tumors (RDR‐C234 and RDR‐A677) were kindly provided by Jean Zhao from Dana‐Farber Cancer Institute (Liu *et al*., 2011). Sources of other reagents were as follows: XAV939 (Tocris, Minneapolis, MN, USA); S31‐201 (SelleckChem, Houston, TX, USA); linsitinib, OSI‐906 (LC Labs, Woburn, MA, USA); LY294002 (Sigma‐Aldrich, St. Louis, MO, USA), and GDC‐0941 (Genentech, San Francisco, CA, USA). *Neu*‐N (c‐erbB2, ERBB2, Her‐2) mice were derived from the colony of Guy *et al*. (1992), bred to homozygosity as verified by Southern blot analysis (Reilly *et al*., 2000), and maintained at Johns Hopkins University.

### 2.2 Site‐directed mutagenesis and cloning of human PIK3CA‐mutant transgenes into MKbpAII vector

The plasmid containing the PIK3CA‐mutant H1047R and E545K sequences were generated by the use of site‐directed mutagenesis kit (Quick Change; Stratagene, La Jolla, CA, USA). Automated design of mutagenic primers for site‐directed mutagenesis was performed by primerx software (Bioinformatics.org).

Primers used were as follows: H1047R forward: 5′‐CAAATGAATGATGCACGTCATGGTGGCTGGAC‐3′, reverse: 5′‐GTCCAGCCACCATGACGTGCATCATTCATTTG‐3′; E545K forward: 5′‐CTCTCTGAAATCACTAAGCAGGAGAAAGATTTTC‐3′, reverse: 5′‐GAAAATCTTTCTCCTGCTTAGTGATTTCAGAGAG‐3′.

Mammary gland‐specific expression of PIK3CA was driven using wild‐type or E545K‐ or H1047R PIK3CA‐mutant genes cloned into the MKbpAII vector containing the mouse mammary tumor virus MMTV‐LTR promoter/enhancer and KCR intron to increase transgene expression (gift from Jeffrey Rosen, Baylor College of Medicine).

### 2.3 HS578T‐PIK3CA‐mutant cell lines

HS578T breast cancer cells were stably transfected with pcDNA3.1‐PIK3CA‐mutant plasmids (H1047R or E545K) using FuGENE 6 (Roche Molecular Systems, Branchburg, NJ, USA) according to the manufacturer's instructions. Successfully transfected cells were selected in culture medium containing 700 μg·mL^−1^ G418 (ThermoFisher Scientific, Waltham, MA, USA) for 4 weeks before the clones were isolated. The clones were pooled and maintained in complete medium containing 400 μg·mL^−1^ of  G418.

### 2.4 Generation, handling, and genotyping of transgenic mice

All animal studies were performed following approval of the Institutional Animal Care and Use Committee of the Johns Hopkins School of Medicine. Transgenic mice were generated by microinjecting the 9.4‐kb fragment of pMMTV‐PIK3CA into the pronucleus of single‐cell embryos isolated from superovulated FVB/N mice by the Transgenic Mouse Core Facility at National Cancer Institute. The transgenic founders from the different PIK3CA‐mutant strains were transferred to Johns Hopkins animal facility. The transgenic F1 generation was crossed with FVB/N mice and the progeny maintained as hemizygous for the PIK3CA‐mutant allele. The genotype of transgenic mice was assessed in tail clip DNA by PCR using MMTV and PIK3CA‐specific primers (forward primer, 5′‐CTGGTCATCATCCTGCCTTT‐3′; reverse primer, 5′‐CCTCACGGAGGCATTCTAAA‐3′). The presence of the respective PIK3CA mutations in the transgenic mouse DNA was confirmed by sequencing.

### 2.5 Determination of transgene copy number and expression

Southern blot analysis and real‐time quantitative PCR (D'Haene *et al*., 2010) were employed to determine the transgene copy number inserted into the founder genome. Briefly, mouse genomic DNA was digested with Bam H1, hybridized with the E545K or H1047R PIK3CA probes, and the membranes were developed using the Odyssey reagent (Licor Biosciences, Lincoln, NE, USA), according to the manufacturer's instructions. For gene expression, real‐time quantitative PCR was conducted using the Maxima SYBR Green/ROX Master Mix (Fermentas, Waltham, MA, USA) per the manufacturer protocol as previously described (Merino *et al*., 2016). Primer sequences will be provided upon request.

### 2.6 Immunohistochemistry (IHC) of mouse tumors

The rabbit antiphosphorylated STAT3 primary antibody (#9131; Cell Signaling, Beverly, MA, USA) was used at 1 : 100 dilution and IHC performed as described (Merino *et al*., 2016).

### 2.7 Histology

5‐μm thick sections of FFPE mouse tumor fragments were stained with hematoxylin and eosin (H&E) and assessed by the pathologist blinded to group of origin.

### 2.8 Whole‐mount analysis

The number 4 (inguinal) mammary glands were dissected, fixed overnight in Carnoy's solution (3 : 1, 95% ethanol and glacial acetic acid), and stained with carmine alum as previously described (Gu *et al*., 2009).

### 2.9 Cell proliferation assay

5 × 10^3^ cells per well were plated in 96‐well plates in triplicate and treated with the indicated drugs, alone and in combination. After 48 h, cell proliferation assay was performed as previously described (Merino *et al*., 2016).

### 2.10 Western blot analysis

Western blotting was performed as previously described (Merino *et al*., 2016) using antibodies to phosphorylated AKT‐Ser (#4058S), phosphorylated GSK3‐β (#9323P), and phosphorylated STAT3 (#9131) from Cell Signaling, Danvers, MA, USA; activated β‐catenin (#05665), STAT3 (#06596), and GAPDH (#MAB374) from Millipore, Billerica, MA, USA; β‐catenin (#138400) and cyclin D1 (#AHF0102) from ThermoFisher Scientific.

### 2.11 Flow cytometry

Single cells were isolated from mammary gland and tumors (Shackleton *et al*., 2006) and stained with CD24‐PE (clone M1/69), 7AAD (BD Pharmingen, San Diego, CA, USA), and CD49f‐APC (clone GoH3; RD Systems). Samples were run on the BD FACSCalibur system (Becton Dickinson, San Antonio, TX, USA), and data were analyzed using flowjo software (FlowJo LLC, Ashland, OR, USA) .

### 2.12 Analysis of TCGA data

The processed RNA‐seq dataset and clinical information of the The Cancer Genome Atlas (TCGA) breast carcinoma (BRCA) database hosted on the Firehose server managed by the Broad Institute (Center, 2015) were used for analysis of correlations of gene expression with disease subtype. PIK3CA mutation and HER2 amplification information was obtained from the cBioPortal interactive analysis platform (Gao *et al*., 2013). Single sample gene set analysis (ssGSEA) (Barbie *et al*., 2009) implemented by the GSVA package (Hanzelmann *et al*., 2013) was used to estimate activation scores of PIK3CA‐related pathways curated by the Molecular Signatures Database (Liberzon *et al*., 2011). Pairwise Welch's corrected *t*‐test with Bonferroni correction for multiple testing was used to calculate significance in differences across groups.

### 2.13 Statistical analysis

mRNA expression, assessed by Q‐PCR, was expressed as mean ± standard errors of mean. Mann–Whitney *U*‐test was performed to assess median of mRNA and protein levels for tumors. Student's *t*‐test was performed to compare cell viability, mRNA, and protein levels in cell lines and expressed as the mean (± SEM). Statistical significance was defined as *P* < 0.05 and calculated using graphpad prism software (GraphPad Software, La Jolla, CA, USA).

## 3 Results

### 3.1 Generation of transgenic mice with mammary gland‐specific overexpression of PIK3CA‐H1047R‐ and ‐E545K‐mutant genes

**Figure 1:**
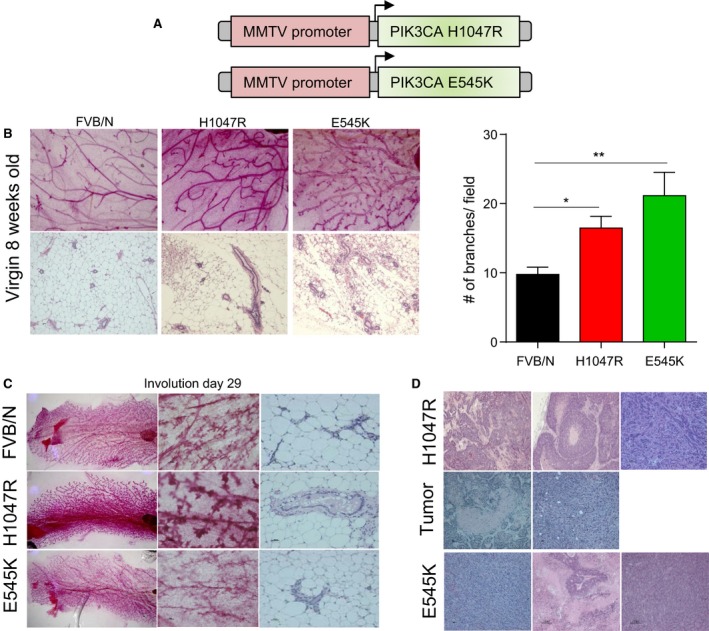
PIK3CA mutations increase mammary gland side branching, delay involution, and induce tumorigenesis. (A) Schema of the constructs used to generate transgenic mice expressing H1047R‐ and E545K‐mutant PIK3CA in the mammary gland. (B) Whole‐mount analysis (upper panel), hematoxylin and eosin (H&E) analysis (lower panel), and quantification of branching in mammary glands from parental FVB/N and mutant PIK3CA mice at 8 weeks of age. (C) Whole‐mount analysis (left panel) and H&E analysis (right panel) of mammary glands from the same group of mice at involution (day 29) stage. (D) Histology of mammary tumors that developed in PIK3CA‐H1047R and ‐E545K mice. Student's *t*‐test, results are expressed as mean ± SEM. *P < 0.05; **P < 0.01; ***P < 0.001.

To examine the role of PIK3CA‐H1047R and ‐E545K mutations during mammary development and their ability to induce mammary tumors, we generated transgenic mice with expression of the PIK3CA‐mutant genes under the control of the mammary epithelial‐specific MMTV promoter (Fig. 1A). The presence of mutant PIK3CA in the transgenic mouse DNA was confirmed by sequencing. Southern blot analysis (Fig. S1A) and quantitative PCR (Fig. S1B) were employed to determine copy numbers of the transgene inserted into the founder genome. As PIK3CA mutations occur on single alleles in the genome, we selected the founders that had a small number of transgene insertions in their genome. In comparison with FVB/N control mice, levels of PIK3CA mRNA (Fig. S1C), protein (Fig. S1D), as well as its downstream effector protein phosphorylated Akt‐Ser (Fig. S1D), were found to be higher in the mammary glands of pregnant transgenic mice. These findings are as predicted because the MMTV promoter is hormonally regulated and is activated in mammary gland during pregnancy and peaks at lactation (Wagner *et al*., 2001).

### 3.2 Mutants of PIK3CA increase side branching, delay involution, and induce tumorigenesis

To examine the effects of PIK3CA mutations on mammary gland development, we performed whole‐mount staining analysis of mammary glands from newly generated PIK3CA‐H1047R and ‐E545K low copy number transgenic mice at different stages of mammary gland development (virgin, pregnancy, and involution). At 8 weeks, the ductal tree of the PIK3CA‐H1047R and ‐E545K transgenic mice had a significantly greater level of branching in comparison with control FVB/N mice (Fig. 1B). On day 13 of pregnancy, there were no significant phenotypic differences in the morphology of the mammary gland among these mice, except a slight increase in branching observed in the H1047R mutants (Fig. S1E). At day 29 of involution, most of the alveolar structures of FVB/N and PIK3CA‐E545K mammary glands collapsed into clusters of epithelial cells and more adipocytes filled the spaces (Fig. 1C). On the other hand, PIK3CA‐H1047R mice showed delayed involution and the alveolar structures were largely intact and filled with milk (Fig. 1C). Using Wnt‐knockout mice, Brisken *et al*. (2000) showed that Wnt proteins function as paracrine factors that operate downstream of progesterone and the progesterone receptor to mediate the process of side branching (Brennan and Brown, 2004). As we observed altered side branching in the mammary glands of PIK3CA‐mutant mice, we investigated whether the Wnt/β‐catenin pathway is induced in the PIK3CA‐H1047R‐ and PIK3CA‐E545K‐mutant mammary glands. We found that mammary glands of PIK3CA‐mutant mice showed an increase in activated β‐catenin (Fig. S1F) and its downstream gene, cyclin D1 (Fig. S1D).

Parous mice with low copy number of PIK3CA‐H1047R and ‐E545K alleles were observed for 2 years to determine whether H1047R and ‐E545K transgenes initiate mammary tumorigenesis. In the PIK3CA‐H1047R strain, tumors arose in 12 (30%) of 40, and in PIK3CA‐E545K strain, tumors arose in 4 (13%) of 30 aged female mice. Tumors were carcinomas with tubular, papillary, and comedo features (Fig. 1D). As previously reported in PIK3CA‐H1047R‐knock‐in mouse models (Tikoo *et al*., 2012; Yuan *et al*., 2013), these tumors arose with long latency (> 12 months). No tumors were observed in the aging wild‐type female FVB/N littermates. PIK3CA‐H1047R tumors expressed estrogen receptor (ER) alpha at levels similar to ER‐positive breast cancer cell lines (Fig. S1G).

### 3.3 Wnt/β‐catenin pathway is activated in PIK3CA‐H1047R‐mutant tumors

Higher levels of β‐catenin expression in the normal glands of PIK3CA‐mutant mice (Fig. S1F), and reports that PIK3CA‐H1047R caused an expansion of the luminal progenitor population (Tikoo *et al*., 2012), led us to hypothesize that the Wnt signaling pathway is likely to be activated in the PIK3CA‐H1047R human breast tumors. We also compared signaling responses between Her2‐ and PIK3CA‐H1047R mouse tumors.

**Figure 2:**
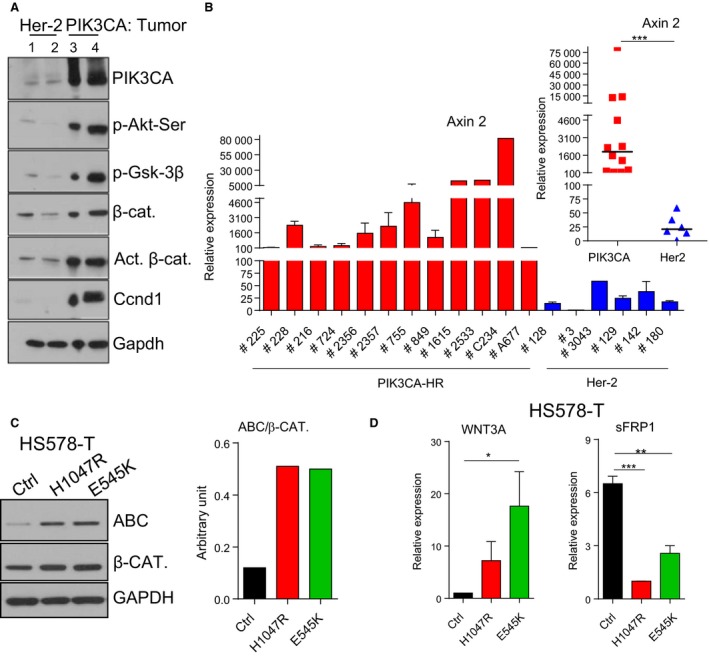
Wnt/β‐catenin pathway is activated in PIK3CA‐H1047R tumors. (A) Western blot analysis of mammary tumors from Her2/neu‐overexpressing and PIK3CA‐H1047R transgenic mice. (B) Quantitative RT‐PCR of Axin 2 expression in PIK3CA‐H1047R (PIK3CA‐HR)‐mutant and Her‐2 mammary tumors. The mean (± SEM) of mRNA in each tumor in triplicate is shown. Mann–Whitney *U*‐test was performed, and the median of mRNA levels for all tumors is shown on the top right. RPL19 was used as housekeeping. (C) Western blot and quantification of activated β‐catenin (ABC) and β‐catenin proteins in human breast cancer cell line HS578T overexpressing either vector control (Ctrl) or PIK3CA‐H1047R and E545K. GAPDH: loading control. (D) Quantitative RT‐PCR of the ligand WNT3A (left) and the pathway negative modulator secreted frizzled‐related protein 1 (sFRP‐1, right) mRNA in HS578T control (Ctrl) or mutant PIK3CA. Student's *t*‐test was performed, and the mean (± SEM) of the triplicate is shown. *P < 0.05; **P < 0.01; ***P < 0.001.

Although PI3K is downstream of Her‐2 signaling, we saw many differences between mammary tumors arising in transgenic mice overexpressing Her‐2 versus PIK3CA‐H1047R, reflecting differences observed between wild‐type and mutant PI3K. PIK3CA‐H1047R mammary tumors showed strong activation of phosphorylated AKT, phosphorylated Gsk3‐β, cyclin D1, and activated β‐catenin compared to tumors from Her‐2 transgenic mice (Fig. 2A). The mRNA expression levels of Axin 2, a transcriptional target of the Wnt/β‐catenin pathway (Jho *et al*., 2002), were higher in PIK3CA‐H1047R‐mutant tumors compared to Her‐2 tumors (Fig. 2B). Axin 2 was also induced in tumor cells derived from recurrent tumors (RDR‐C234 and RDR‐A677) from the H1047R‐doxycycline‐inducible mouse model (Liu *et al*., 2011) (Fig. 2B). Consistent with predicted consequences of activation of the Wnt/β‐catenin pathway, single cells isolated from PIK3CA‐H1047R tumors contained a larger CD24med/CD49fhi stem cell population (63%) compared to normal mammary glands (12% at 2 months and 20% at pregnancy) (Fig. S2). Although it was reported that PIK3CA‐H1047R caused an expansion of the luminal progenitor population (Tikoo *et al*., 2012), we did not observe a difference in the CD24med/CD49fhi stem cell population between FVBN and PIK3CA‐H1047R mammary glands.

To extend these findings in mouse mammary tumors to human tumor cells, we overexpressed PIK3CA‐H1047R‐ and PIK3CA‐E545K‐mutant genes in the human breast cancer cell line, HS578T. Human breast cancer cells expressing mutant PIK3CA also showed activation of the WNT/β‐catenin pathway, which was detected as an increase in activated β‐catenin (ABC) protein (Fig. 2C), the ligand WNT3A mRNA (Fig. 2D, left panel), and a decrease in mRNA expression of the negative modulator of the pathway, the secreted frizzled‐related protein 1 (sFRP‐1) (Fig. 2D, right panel).

### 3.4 STAT3 is activated in PIK3CA‐H1047R tumors

An IL‐6‐STAT3 loop was shown to mediate resistance to PI3K inhibitors by inducing epithelial–mesenchymal transition (EMT) and cancer stem cell expansion in human breast cancer cells (Yang *et al*., 2014). Therefore, we investigated whether Il6‐Stat3 signaling is induced in H1047R‐mouse tumors and could be utilized as a therapeutic target in combination with PI3K inhibitors.

**Figure 3:**
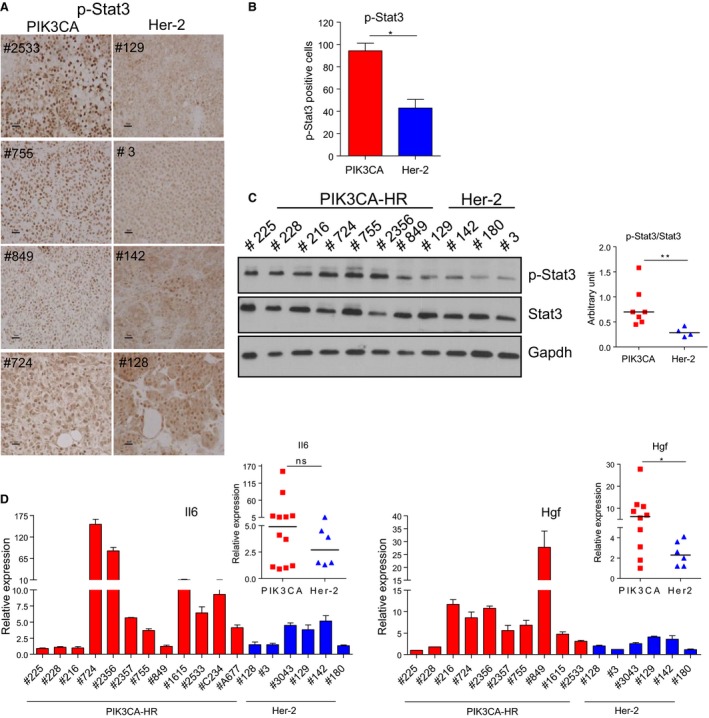
Stat3 is activated in PIK3CA‐H1047R tumors. (A) Immunohistochemistry analysis and (B) Student's *t*‐test quantification of phosphorylated Stat3 protein in PIK3CA and Her‐2 tumors. (C) Western blot (left panel) and Mann–Whitney *U‐*test quantification (right panel) of phosphorylated‐Stat3 and Stat3 in PIK3CA‐H1047R and Her‐2 tumors. Gapdh: loading control. Quantitative RT‐PCR of (D) interleukin 6 (Il6) and (E) hepatocyte growth factor (Hgf) expression in PIK3CA‐H1047R (PIK3CA‐HR)‐mutant and Her‐2 tumors. RPL19 was used as a housekeeping gene. The mean (± SEM) of mRNA in each tumor in triplicate is shown. Mann–Whitney *U*‐test was performed, and the median of mRNA levels for all tumors is shown on the right. *P < 0.05; **P < 0.01; ***P < 0.001.

Phosphorylated Stat3 expression was higher in the mouse mammary tumors from PIK3CA‐H1047R mice in comparison with tumors from Her‐2/neu transgenic mice, as determined by IHC (Fig. 3A,B) and western blot analysis (Fig. 3C). Upstream of Stat3, Il6 tended to be highly expressed (Fig. 3D) and hepatocyte growth factor (Hgf) is significantly highly expressed (Fig. 3E) in mammary tumors arising in PIK3CA‐H1047R in comparison with Her‐2 transgenic mice.

### 3.5 Insulin‐like growth factor (IGF) is activated in PIK3CA‐H1047R tumors

**Figure 4:**
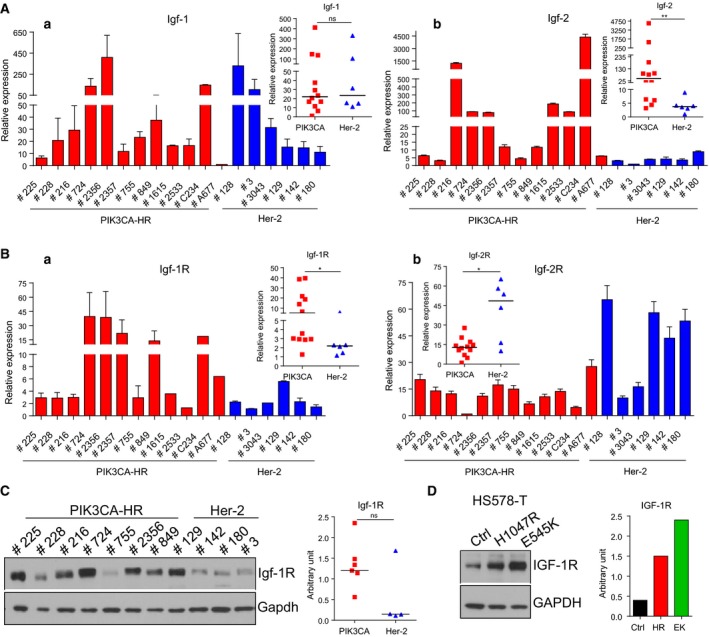
Insulin‐like growth factor (Igf‐1) is activated in PIK3CA‐H1047R tumors. In PIK3CA‐H1047R (PIK3CA‐HR)‐mutant and Her‐2 tumors, quantitative RT‐PCR of (A) the ligands Igf‐1 (a) and Igf‐2 (b), and (B) the receptors Igf‐1R (a) and Igf‐2R (b). RPL19 was used as a housekeeping gene. The mean (± SEM) of mRNA in each tumor in triplicate is shown. Mann–Whitney *U*‐test was performed, and the median of mRNA levels for all tumors is shown on the top. Western blot and quantification of IGF‐1R (C) in PIK3CA‐H1047R‐mutant and Her‐2 mouse tumors, and (D) in HS578T human breast cancer cells overexpressing vector control (Ctrl) or PIK3CA‐H1047R and ‐E545K mutants. Mann–Whitney *U*‐test was performed, and the median of protein levels for all tumors is shown. *P < 0.05; **P < 0.01; ***P < 0.001.

The activation of insulin‐like growth factor 1 receptor (Igf‐1R) and downstream PI3K pathway has been implicated in resistance to PI3K inhibitors (Leroy *et al*., 2016). Although there was no difference in Igf‐1 mRNA levels (Fig. 4A, a), Igf‐2 mRNA was significantly higher in PIK3CA‐H1047R mammary tumors in comparison with Her‐2 tumors (Fig. 4A, b). PIK3CA‐H1047R tumors also showed higher mRNA levels of Igf‐1R (Fig. 4B, a) and lower levels of Igf‐2R (Fig. 4B, b) in comparison with Her‐2 tumors. Igf‐1R protein levels tended to be higher in PIK3CA‐H1047R tumors in comparison with Her‐2 tumors (Fig. 4C). HS578T human breast cancer cell lines overexpressing the H1047R showed threefold, and E545K mutants showed fivefold higher IGF‐1R levels than the parental HS578T cells (Fig. 4D).

### 3.6 PIK3CA‐mutant tumor cells are targeted by anti‐Wnt, anti‐STAT3, and anti‐IGF‐1R drugs in combination with PI3K inhibitors

We assembled a panel of cell lines from our PIK3CA‐H1047R mouse mammary tumors, generated human HS578T‐H1047R and ‐E545K breast cancer cell lines, and obtained cell lines derived from recurrent H1047R mouse tumors (RDR‐C234 and RDR‐A677) (Liu *et al*., 2011). We evaluated their sensitivity to inhibitors of Wnt/β‐catenin (XAV939), STAT3 (S31‐201), and IGF‐1R (OSI‐906) alone, or in combination with the PI3K inhibitor, LY294002.

**Figure 5:**
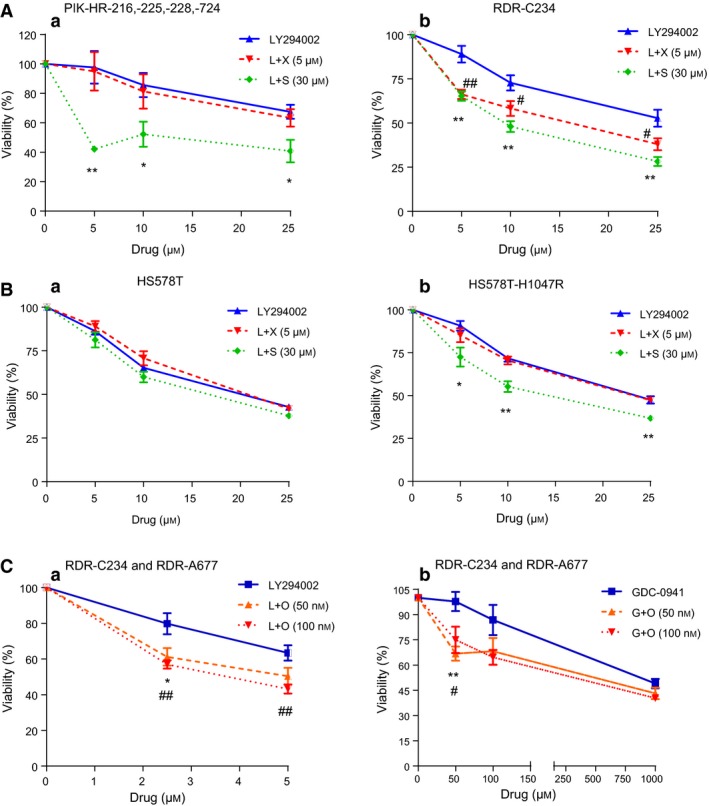
PIK3CA‐mutant tumor cells are targeted with Wnt, STAT3, and IGF‐1R drugs in combination with PI3K inhibitors. Cell growth inhibition upon treatment for 48 h with the PI3Ki, LY294002 alone or in combination with an inhibitor of β‐catenin, XAV939 (X, 5 μm), or STAT3, S31‐201 (S, 30 μm). P values (*) of L+S and (#) of L+X, in relation to LY294002. (A) Mammary tumor cell lines from (a) H1047R‐MMTV transgenic (PIK‐HR‐216, ‐225, ‐228, ‐724) and (b) doxycycline‐inducible H1047R (RDR‐C234) mice. (B) Human breast cancer cell line (a) HS578T or (b) HS578T‐H1047R cells. (C) Two PIK3CA‐H1047R tumor cell lines derived from doxycycline‐inducible H1047R mouse tumors (RDR‐A677 and RDR‐C234) were treated (a) for 48 h with the PI3K inhibitors LY294002 (2.5 and 5 μm) alone and (b) GDC‐0941(G, 0.05, 0.1, and 1 μm) alone, or in combination with an inhibitor of IGF‐1R, OSI‐906 (O, 50 and 100 nm). P values (*) of L+O or G+O (O, 50 nm) and (#) of L+O or G+O (O, 100 nm), in relation to LY294002 or GDC‐0941, are shown. Student's *t*‐test was performed, and the mean (± SEM) of the quadruplicate is shown. *P < 0.05; **P < 0.01; ***P < 0.001.

Treatment of PIK3CA‐H1047R mouse tumor cells and RDR‐C234 with XAV939 (1, 5, and 10 μm) or S31‐201 (3, 15, and 30 μm) alone had no effect on cytotoxicity (Fig. S3), while the cells were sensitive to the combination of PI3K and STAT3 inhibitors (Fig. 5A a, b). RDR‐C234 cells were also sensitive to PI3Ki in combination with Wnt/β‐catenin inhibitors (Fig. 5A, b).

There was no increase in the cytotoxic effect of the combination therapies in comparison with PI3K inhibition alone on parental HS‐578T breast cancer cells (Fig. 5B, a). In contrast, HS578T cells that overexpressed PIK3CA‐H1047R responded more efficiently to combinations of PI3K and STAT3 inhibitors compared to single agents (Fig. 5B, b). Similar to cell lines derived from our PIK3CA‐H1047R transgenic mice tumors (Fig. 5A, a) the combination of PI3K and Wnt/β‐catenin inhibitors did not improve the cytotoxicity achieved by the PI3Ki alone (Fig. 5B, b). These results suggest that combination of PI3Ki with STAT3 inhibitor is more effective than combination of PI3Ki with the Wnt/β‐catenin inhibitor. In addition, H1047R mouse tumor cells, RDR‐C234 and RDR‐A677, derived from recurrent tumors, responded more efficiently to a combination of inhibitors of PI3K (both LY294002 and GDC‐0941) and IGF‐1R (OSI‐906) in comparison with single treatments (Fig. 5C). These results with mouse and human cells containing the PIK3CA hotspot mutation H1047R suggest that targeting specific pathways that are additionally activated in these tumors potentiates the killing effect of PI3Ki and may overcome drug resistance.

### 3.7 STAT3, β‐catenin, and IGF pathways are induced in human PIK3CA tumors

**Figure 6:**
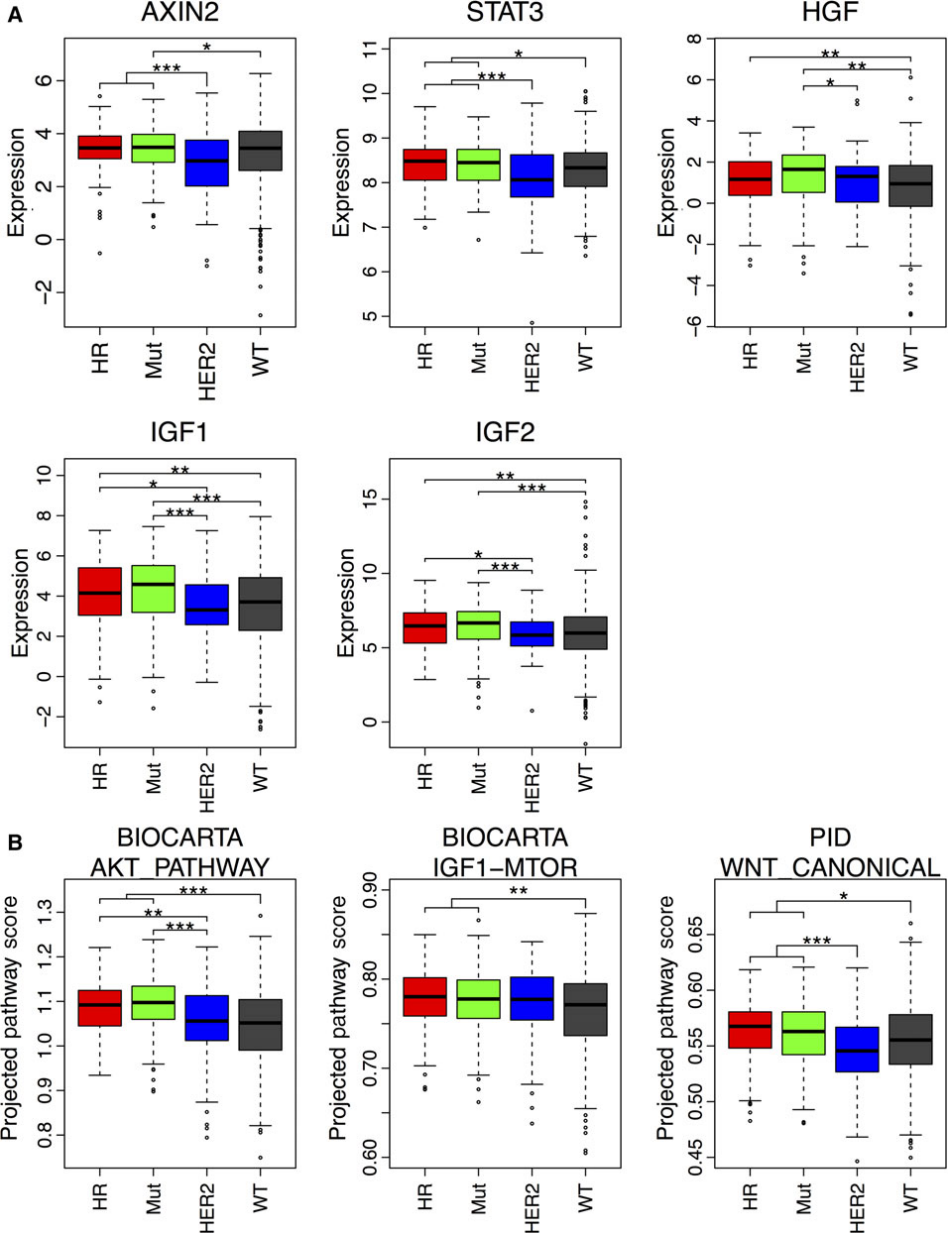
STAT3, β‐catenin, and IGF pathways are induced in human PIK3CA tumors. (A) Expression of mutated PIK3CA‐related genes in 965 primary luminal breast tumors using The Cancer Genome Atlas (Center) RNA‐seq dataset. (B) Pathway score estimation of mutated PIK3CA‐related signaling pathways in the same tumor set using the ssGSEA (single sample gene set analysis) method. PIK3CA‐H1047R (HR,* n* = 125), other PIK3CA mutants (Mut, *n* = 188), including E545K and E542K, Her2 (*n* = 118), and non‐PIK3CA‐mutant (wild‐type, wt, *n* = 534) breast tumors. Pairwise Welch's corrected *t*‐test with Bonferroni correction for multiple testing was used. *P < 0.05; **P < 0.01; ***P < 0.001.

We next evaluated whether the pathways activated in the mutated PIK3CA mouse tumors involved in the cell killing effect in combination with the PI3Ki are also regulated in luminal human tumors containing the PIK3CA hotspot mutations. Analysis of 965 primary breast tumors from TCGA RNA‐seq dataset showed that breast tumors harboring H1047R and other PIK3CA mutations (including E545K and E542K) have significant upregulation of AXIN2, HGF, STAT3, IGF‐1, and IGF‐2 mRNA compared with PIK3CA‐wild‐type and HER‐2‐amplified tumors (Fig. 6A). Estimated using the ssGSEA (single sample gene set analysis) method (Barbie *et al*., 2009), PIK3CA‐mutant breast tumors showed activated AKT, IGF1‐mTOR, and WNT canonical signaling pathways compared to PIK3CA‐wild‐type and HER2‐overexpressing tumors (Fig. 6B).

Thus, our comprehensive molecular analysis of PIK3CA‐H1047R mouse and *in silico* analysis of human tumors revealed activation of Wnt/β‐catenin, STAT3, and IGF‐1R pathways. Therefore, combined targeting of pathways altered in PIK3CA‐mutant tumors offers the potential to improve treatment regimens with PI3K inhibitors.

## 4 Discussion

PIK3CA, the gene encoding the catalytic subunit p110a of PI3K, is an excellent target for therapy. Since their discovery, PI3K inhibitors have been developed but have experienced limited success in the clinic. One of the underlying reasons is the possible reliance of the tumors on activated compensatory pathways occurring upon PI3K blockade. To decipher other pathways that are activated in PIK3CA‐mutant tumors and with a goal of developing improved treatment regimens for mutant tumors, we generated transgenic mouse models with mammary gland‐specific overexpression of the PIK3CA hot spot mutations, H1047R and E545K. We have identified key alternate pathways active in mutant tumors and have demonstrated effective therapy regimens that use combinations of PI3K‐i and other pathway‐specific drugs.

Characterization of the transgenic models showed that mutant mice have increased mammary ductal branching compared to control FVB/N, and H1047R mutants displayed delayed involution (Fig. 1B,C). Mouse models of PIK3CA‐H1047R transgenic mice showed abnormalities in mammary gland development such as an increase in mammary ductal side branching (Adams *et al*., 2011; Tikoo *et al*., 2012). Further, transgenic mice with alteration in PI3K pathway, such as AKT activation (Schwertfeger *et al*., 2001), PIK3CA membrane localization (Renner *et al*., 2008), and PIK3CA‐H1047R (Meyer *et al*., 2011), showed delayed involution, attributed to a reduced number of apoptotic cells. Transgenic mice with loss of PTEN exhibited excessive ductal branching, delayed involution, and severely reduced apoptosis (Jho *et al*., 2002). In PIK3CA‐mutant mice generated by us, we observed that the PIK3CA‐mutant glands have an increase in activated β‐catenin and the downstream cyclin D1. The phenotypes observed in the PIK3CA‐mutant mice were in line with the known role of Wnt signaling in ductal branching (Brennan and Brown, 2004). We observed mammary tumors at the lower end of the frequencies (range 25–100%) as reported by others in the PIK3CA‐H1047R mice (Adams *et al*., 2011; Meyer *et al*., 2011; Tikoo *et al*., 2012; Yuan *et al*., 2013). Tumor incidence was possibly proportional to the expression levels of the mutant protein. We also generated PI3KCA‐E545K transgenic mice. Here again, the incidence was low – 4 (13%) of 30 PIK3CA‐E545K female mice developed mammary tumors. As previously reported in PIK3CA‐H1047R‐knock‐in mouse models (Tikoo *et al*., 2012; Yuan *et al*., 2013) and PIK3CA‐E545K (Meyer *et al*., 2011), we observed that PIK3CA‐H1047R and ‐E545K tumors arose with long latency (> 12 months) (Fig. 1D). It was previously reported that expression of PIK3CA‐mutant H1047R induces heterogeneous tumors (Meyer *et al*., 2011), an observation in line with our own findings of differing histologies in the PIK3CA‐H1047 tumors. Expression of PIK3CA‐mutant E545K in the mouse mammary gland was also shown to induce heterogeneous tumors although it is less potently tumorigenic compared to mutant H1047R (Meyer *et al*., 2013). Also, similar to others (Koren and Bentires‐Alj, 2013), we observed that PIK3CA‐H1047R tumors are ER alpha positive (Fig. S1G).

Following molecular characterization of PIK3CA‐H1047R tumors, we identified different pathways induced in the mutant tumors in comparison with tumors which have an activated, but wild‐type PI3K pathway, such as mammary tumors from the MMTV‐Her2 mice. The Wnt/β‐catenin (Fig. 2) and Stat3 (Fig. 3) pathways were activated in PIK3CA‐H1047R breast tumors and human breast cancer cell line overexpressing mutant PIK3CA. Consistent with this finding, gene expression profiling of PIK3CA‐mutant, ERα‐positive breast cancer revealed activation of the Wnt and Jak/STAT signaling pathway (Cizkova *et al*., 2010). In addition, PTEN deletion and PI3K activation were shown to cooperate with Wnt1 to induce mammary tumors (Li *et al*., 2001). Combinations of PI3K/mTOR and JAK2/STAT5 inhibitors effectively target triple‐negative breast cancer (Britschgi *et al*., 2012). Stat3 signaling was shown to be required for PI3K‐mediated transformation of mouse fibroblast (Hart *et al*., 2011). An IL‐6‐STAT3 loop was shown to mediate resistance to PI3K inhibitors by inducing EMT and cancer stem cell expansion in human breast cancer cells (Yang *et al*., 2014). In fact, Il6 and Hgf (Fig. 3D,E), activators of Stat3, and the stem cell population (Fig. S2) were induced in PIK3CA‐H1047R tumors. Recently, it was described that PIK3CA‐H1047R induces multipotency and multilineage mammary tumors (Koren *et al*., 2015; Van Keymeulen *et al*., 2015).

Another interesting observation was that the expression of insulin‐like growth factor 2 (Igf2) and Igf‐1R was significantly higher, while expression of Igf‐2R, which degrades IGF‐2 (Brown *et al*., 2009), was lower in H1047R tumors (Fig. 4). This finding is consistent with previous observations that Igf‐2R promoted endocytosis and lysosomal degradation of Igf‐2, thereby antagonizing its action and acting as a tumor suppressor protein (Brown *et al*., 2009). These findings are also in accordance with previous reports, which showed that activation of PI3K pathway, through reduction in PTEN in breast tumor cells, is associated with increased proliferation in response to IGF‐2 (Church *et al*., 2012). In addition, a correlation between IGF‐1R and downstream PI3K pathway activation was reported in drug resistance (Fox *et al*., 2011; Gallardo *et al*., 2012; Leroy *et al*., 2016), and IGF‐1R and PI3K dual treatments act synergistically in targeting breast cancer cells (Ayub *et al*., 2015).

Here, we have demonstrated that the use of specific inhibitors to the pathways which we identified as induced in PIK3CA‐mutant tumors, such as STAT3, β‐catenin, and IGF‐1R, potentiates the cytotoxic effect of PI3K inhibitor on mutant PIK3CA tumor cells (Fig. 5). In addition, we showed that H1047R and other PIK3CA mutants, including E545K and E542K containing BRCAs, have significant upregulation of several genes/pathways identified in the mouse PIK3CA‐mutant tumors, when compared to human PIK3CA‐wild‐type and HER‐2‐positive tumors (Fig. 6), suggesting the translational potential of our findings in this paper using model systems.

## 5 Conclusions

In summary, by generating transgenic mice expressing two hotspot mutants of PIK3CA in the mammary glands and through detailed characterization of their tumors, we have identified activation of Wnt/β‐catenin, Stat3, and Igf‐1R pathways. We have shown that the combined targeting of these additional pathways altered in PIK3CA‐mutant tumors may improve the success of treatment regimens that include PI3K inhibitors (Fig. S4).

## Author contributions

VM, SC, XL, and SS were responsible for the conception and experimental design. VM, SC, XL, SP, KJ, QC, and CZ performed the experiments. VM, SC, XL, DP, AR, and SS acquired the data. VM, SC, XL, DP, AR, CZ, and SS conducted analysis and interpretation of data. VM, SC, and SS wrote and revised the manuscript. SS was responsible for study supervision.

## Supporting information


**Fig. S1.** Determination of PIK3CA mutant transgene copy number and expression, and characterization of mutant normal mammary gland.

**Fig. S2.** Stem cell population in PIK3CA‐H1047R normal mammary glands and tumors.

**Fig. S3.** PIK3CA mutant tumor cells are targeted with Wnt, STAT3 and IGF‐1R drugs in combination with PI3K inhibitors.

**Fig. S4.** Schematic representation of signaling pathways activated in PIK3CA mutant breast tumor cells.
