# Supplementary figures and images for: Inhibitors of STAT3, β‐catenin, and IGF‐1R sensitize mouse PIK3CA‐mutant breast cancer to PI3K inhibitors
Source: Mol Oncol. 2017 Apr 6;11(5):552–66. doi: 10.1002/1878-0261.12053 (PMC5527464; doi:10.1002/1878-0261.12053)

## Slide 1
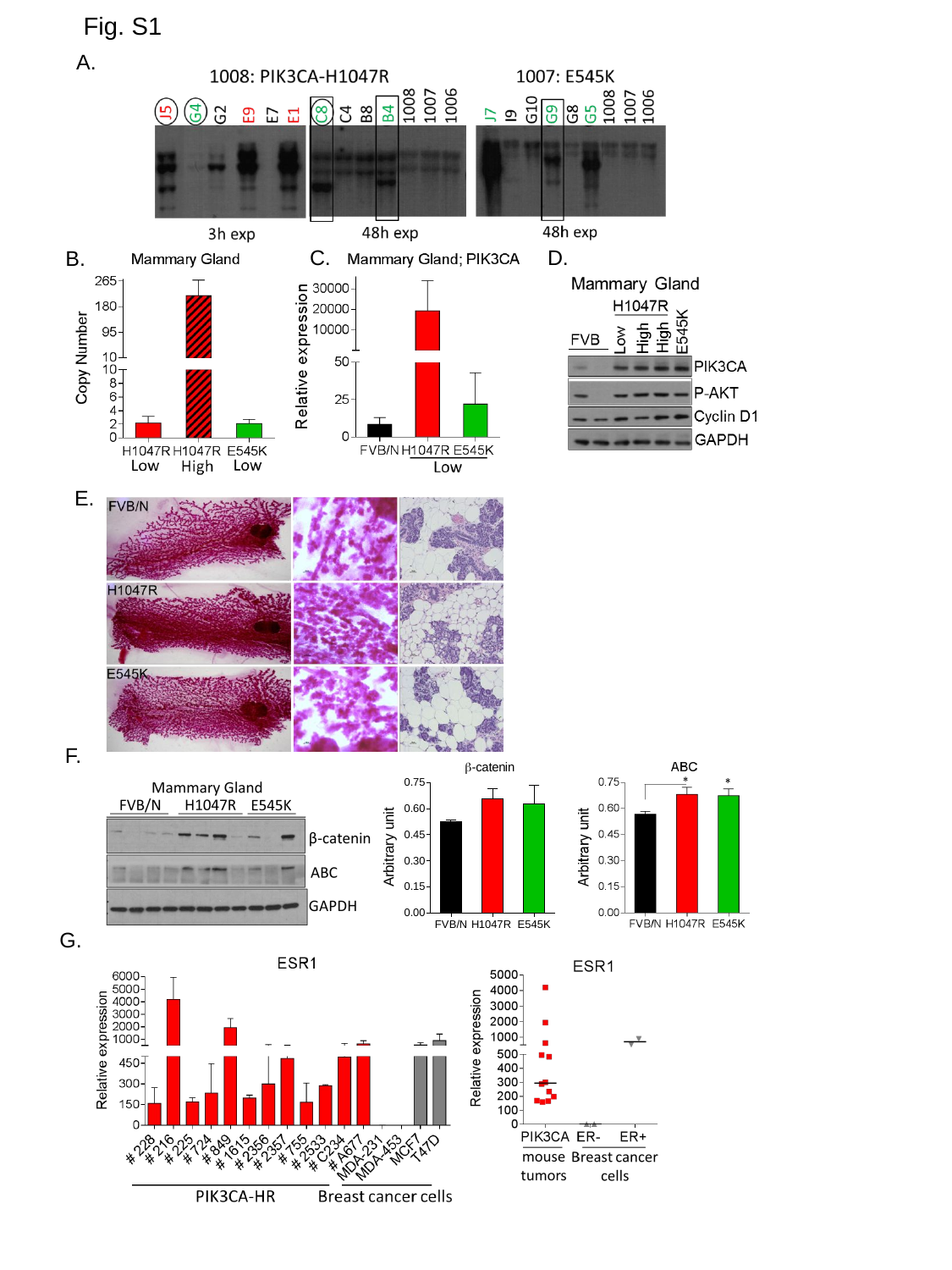

Fig. S1
A.
D.
C.
B.
E.
F.
G.

Supplement: Supplementary file 1 — Fig. S1. Determination of PIK3CA mutant transgene copy number and expression, and characterization of mutant normal mammary gland. [file MOL2-11-552-s001.pptx]

## Slide 1
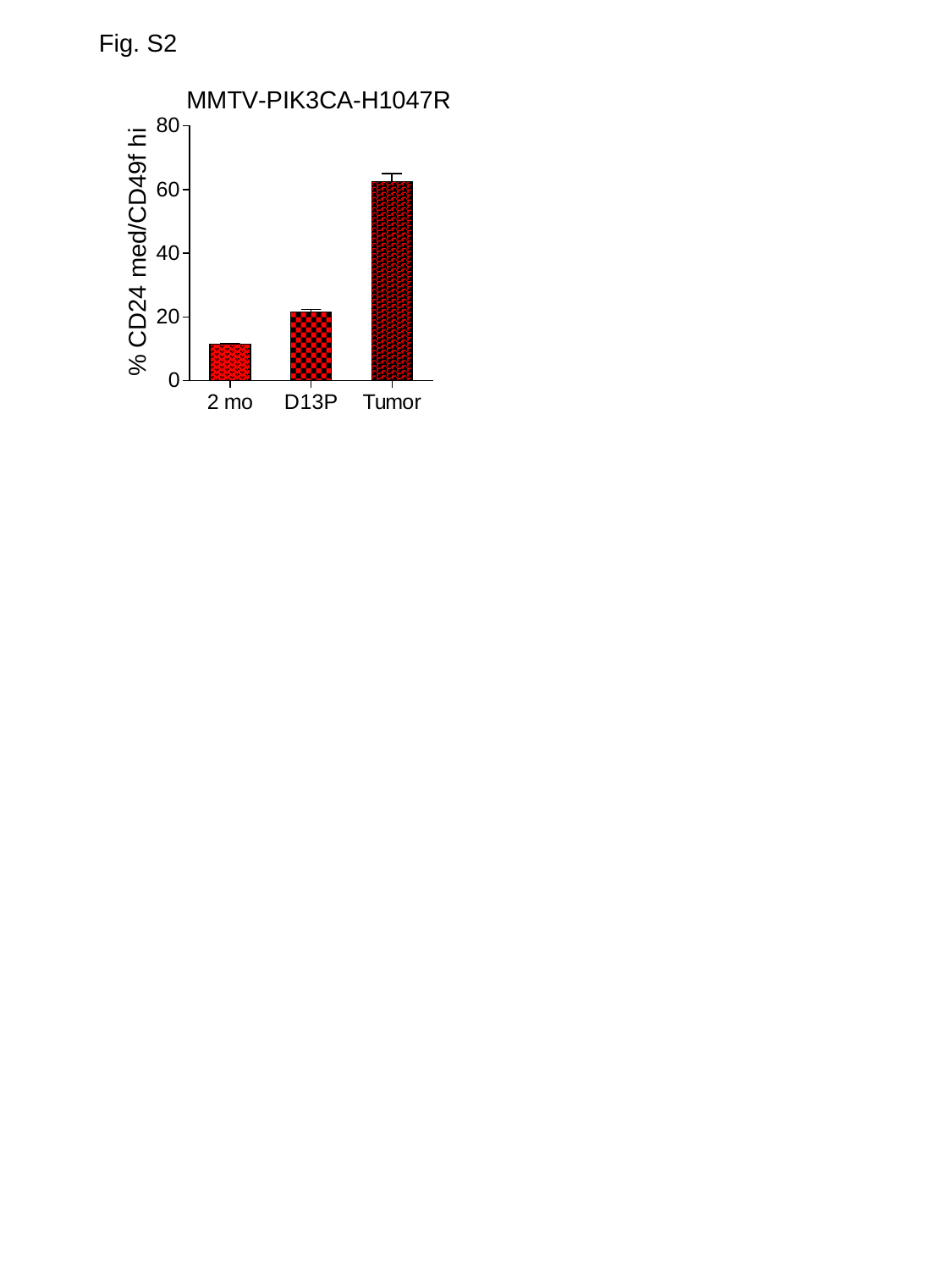

Fig. S2

Supplement: Supplementary file 2 — Fig. S2. Stem cell population in PIK3CA‐H1047R normal mammary glands and tumors. [file MOL2-11-552-s002.pptx]

## Slide 1
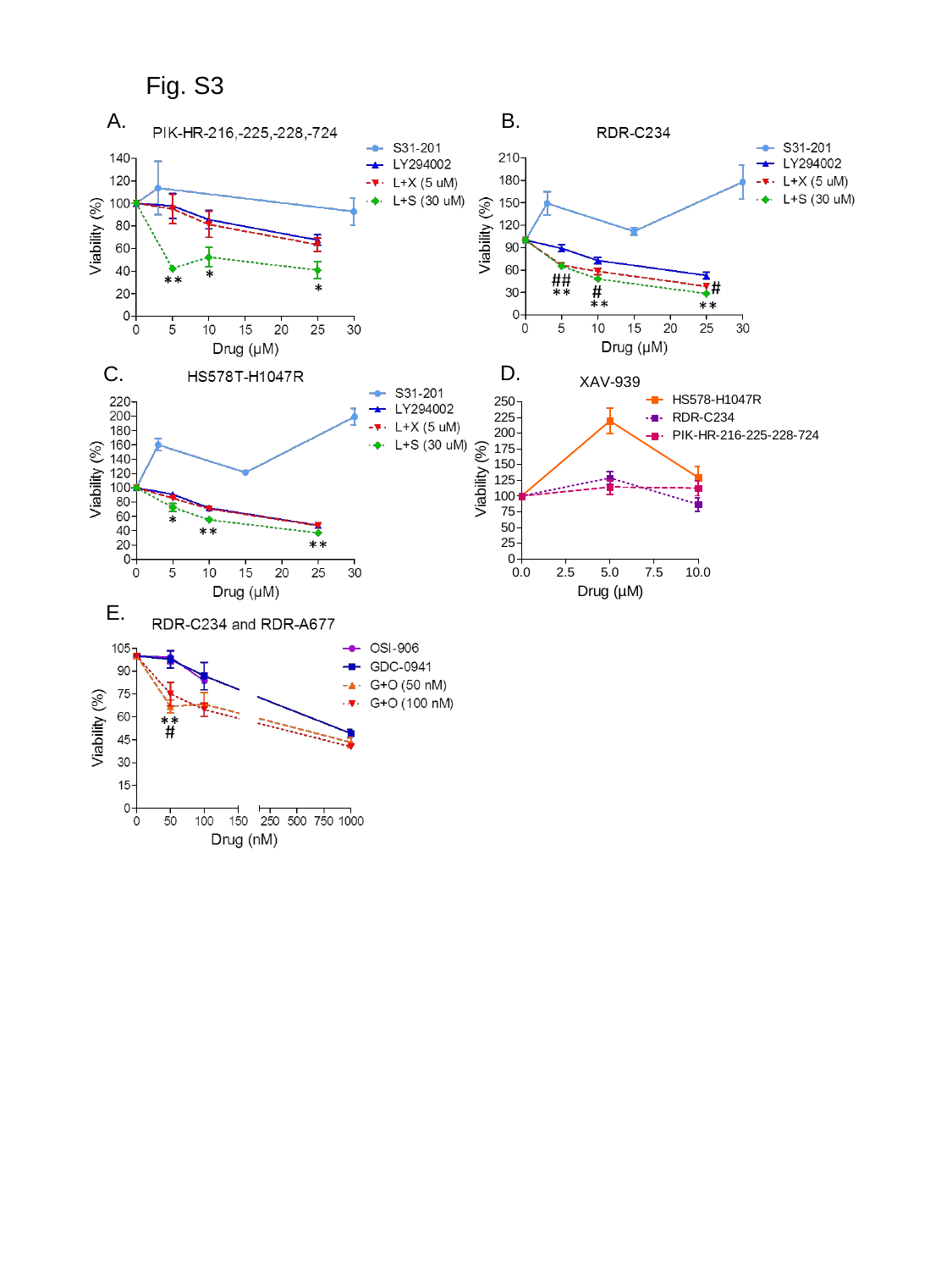

Fig. S3
A.
B.
D.
C.
E.

Supplement: Supplementary file 3 — Fig. S3. PIK3CA mutant tumor cells are targeted with Wnt, STAT3 and IGF‐1R drugs in combination with PI3K inhibitors. [file MOL2-11-552-s003.pptx]

## Slide 1
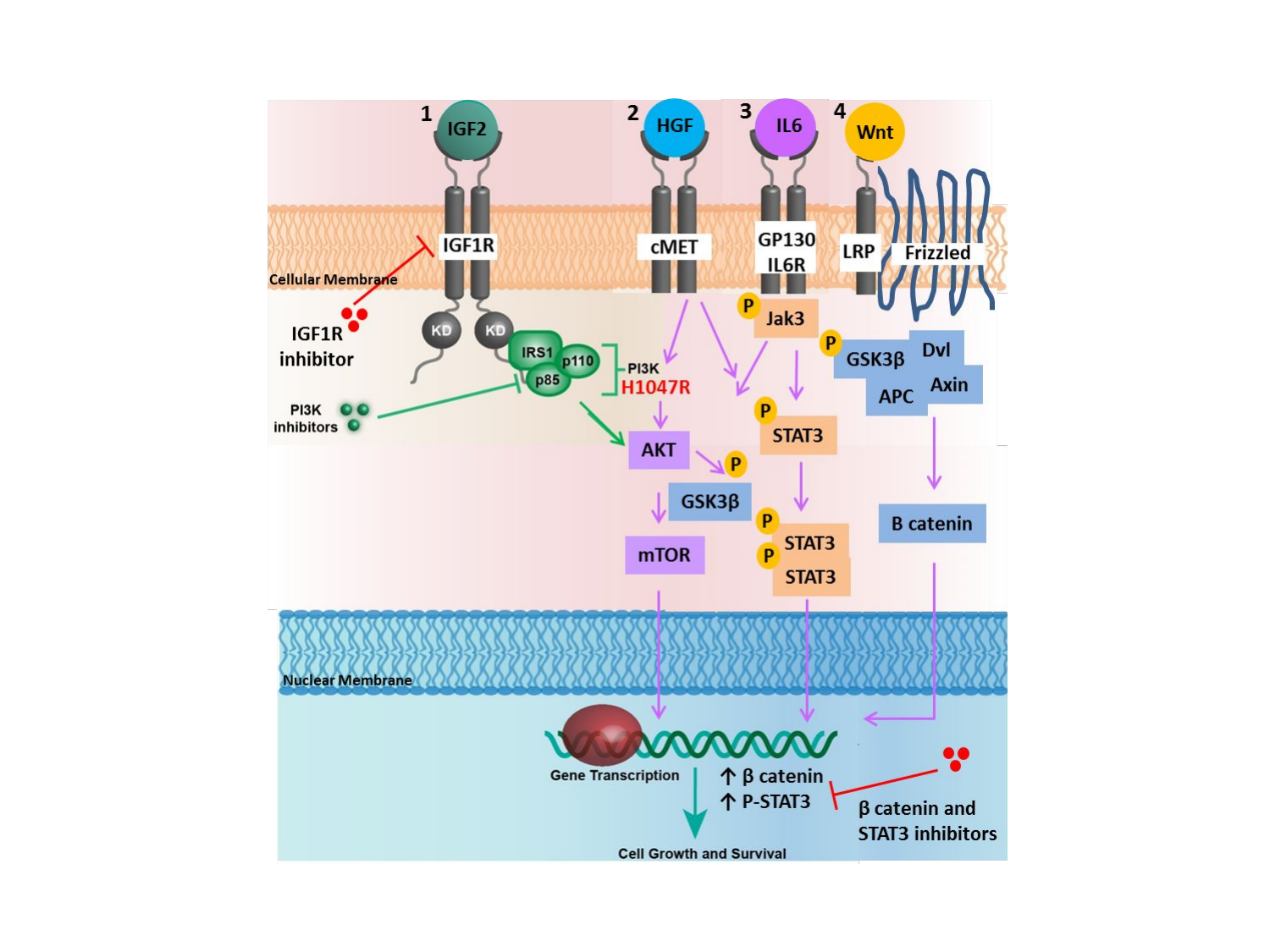

Supplement: Supplementary file 4 — Fig. S4. Schematic representation of signaling pathways activated in PIK3CA mutant breast tumor cells. [file MOL2-11-552-s004.pptx]
